# Chronic bryostatin-1 rescues autistic and cognitive phenotypes in the fragile X mice

**DOI:** 10.1038/s41598-020-74848-6

**Published:** 2020-10-22

**Authors:** Patricia Cogram, Daniel L. Alkon, David Crockford, Robert M. J. Deacon, Michael J. Hurley, Francisco Altimiras, Miao-Kun Sun, Michael Tranfaglia

**Affiliations:** 1 FRAXA-DVI, FRAXA, Santiago, Chile; 2 IEB, Faculty of Science, University of Chile, Santiago, Chile; 3 Neurotrope Bioscience Inc., New York, USA; 4 Neuroimmunology, Biological Sciences, Faculty of Environmental and Life Sciences, University of Southampton, Southampton, UK; 5 Faculty of Engineering, Pontificia Universidad Católica de Valparaíso, Valparaíso, Chile; 6 Faculty of Engineering and Business, Universidad de las Américas, Santiago, Chile; 7 FRAXA Research Foundation, Newburyport, MA USA

**Keywords:** Drug discovery, Autism spectrum disorders

## Abstract

Fragile X syndrome (FXS), an X-chromosome linked intellectual disability, is the leading monogenetic cause of autism spectrum disorder (ASD), a neurodevelopmental condition that currently has no specific drug treatment. Building upon the demonstrated therapeutic effects on spatial memory of bryostatin-1, a relatively specific activator of protein kinase C (PKC)ε, (also of PKCα) on impaired synaptic plasticity/maturation and spatial learning and memory in FXS mice, we investigated whether bryostatin-1 might affect the autistic phenotypes and other behaviors, including open field activity, activities of daily living (nesting and marble burying), at the effective therapeutic dose for spatial memory deficits. Further evaluation included other non-spatial learning and memory tasks. Interestingly, a short period of treatment (5 weeks) only produced very limited or no therapeutic effects on the autistic and cognitive phenotypes in the *Fmr1* KO2 mice, while a longer treatment (13 weeks) with the same dose of bryostatin-1 effectively rescued the autistic and non-spatial learning deficit cognitive phenotypes. It is possible that longer-term treatment would result in further improvement in these fragile X phenotypes. This effect is clearly different from other treatment strategies tested to date, in that the drug shows little acute effect, but strong long-term effects. It also shows no evidence of tolerance, which has been a problem with other drug classes (mGluR5 antagonists, GABA-A and -B agonists). The results strongly suggest that, at appropriate dosing and therapeutic period, chronic bryostatin-1 may have great therapeutic value for both ASD and FXS.

## Introduction

Fragile X Syndrome (FXS), the most common inherited intellectual disability and the leading mono-genetic cause of autism spectrum disorder (ASD)^1,2^, is a neurodevelopmental condition due to a CGG trinucleotide expansion in the fragile X mental retardation 1 (*Fmr1*) gene locus Xq27.3. The expansion leads to a hyper-methylated region in the gene promoter that silences it, thereby lowering the expression level of the fragile X mental retardation (FMRP) protein. FMRP is involved in direct interaction with RNA-editing enzymes^3^ and with multiple mRNAs to regulate their translation, many of which play essential roles in the regulation of synaptic plasticity and synaptic maturation^4^. The lack of FMRP thus results in a global bias for hypo-editing in ASD brains^3^ and dysregulation of synaptic functions and synaptic maturation, resulting from dysregulated brain architecture and synaptic pathways^5^. Although FXS is described commonly as a neurodevelopmental disorder, patients with FXS suffer lifelong cognitive deficits, hyperactivity, depression, disturbance in natural behaviors, and exhibit autistic behavior^6,7^. Unfortunately, the disorder has no specific cure or effective treatment. Several promising preclinical strategies have failed to yield successful outcomes^8–10^, due to either major side effects or the lack of effective improvement of the FXS phenotypes. A recent study has also reported that chronic rapamycin, a mammalian target of rapamycin pathway (mTORC1) inhibitor, does not reverse the behavioral phenotypes in the *Fmr1* knockout mice (*Fmr1* KO2 mice)^11^, suggesting that modulation of the mTOR pathway, hitherto one of the leading therapeutic targets^12^, is not an effective treatment strategy.

Bryostatin-1 is a highly potent and relatively specific activator of protein kinase C (PKC) ε (also of PKCα)^13–15^. It possesses a pharmacological profile that includes rapid mGluR desensitization, synaptogenesis and synaptic maturation/repairing^13,16^. In young and adult fragile X mice, its chronic administration has been found to rescue synaptic and cognitive functions^17,18^, most likely through restoring the experience-dependent maturation of the synapses and circuits^19^. Interestingly, FXS and ASD exhibit common features in genetic makeup and abnormalities of brain circuitry and synapses^5^. Disruptions in experience-dependent maturation of circuits and synapses are also responsible for the autistic behavior^20,21^. We therefore evaluated, in this study, whether chronic bryostatin-1 might also rescue autistic and behavioral phenotypes in *Fmr1* KO2 mice. The aim of the study was to show the chronic effect of bryostatin 1 on ameliorating activities of daily living, habituation and learning and memory in *Fmr1* KO2 mice. Previous treatment strategies tested to date based on mGluR5 antagonists, and GABA-A and -B agonists show showed tolerance. Consequently, in consideration in planning clinical trials, we aimed to evaluate tolerance of long term treatment with bryostatin 1 in *Fmr1* KO2 mice.

## Materials and methods

### Animals and drug treatments

#### Animals

In this study, the mice used were the *Fmr1* KO2 and wild-type (WT) littermates generated on a C57BL/6J background and repeatedly backcrossed onto a C57BL/6J background for more than eight generations. The mice were provided by Professor David Nelson from Baylor College and FRAXA Research Foundation, Massachusetts, USA.

The *Fmr1* KO2 mice were generated by deletion of the promoter and first exon of *Fmr1*^22^. The *Fmr1* KO2 mice are both, protein and mRNA null. *Fmr1* KO2 mice, like Fmr1 KO mice, recapitulate behavioral symptoms observed in humans with FXS, including hyperactivity, repetitive behaviors and deficits in learning and memory^30^.

The mice were housed in 4–5 per cage groups of the same genotype in a temperature- (21 ± 1 °C) and humidity-controlled room with a 12-h light–dark cycle (lights on 7 a.m.–7 p.m.). Food and water were available ad libitum. Mice were housed in commercial plastic cages on a ventilated rack system.

Experiments were conducted in line with the requirements of the UK Animals (Scientific Procedures) Act, 1986. All procedures for animal maintenance and experimentation were approved and followed the recommendations of the ethics committee of the Institute of Ecology and Biodiversity (IEB), Faculty of Sciences of the University of Chile, and complied with Chilean regulations.

Behavioral testing was conducted during the light phase in 2-month-old male mice, 10 mice per treatment group. All experiments were conducted with experimenter blind to genotype and drug treatment. Mice were tested in one behavioral task on each experimental day and each behavioral test was separated by 3 days. Prior to the behavioral testing mice were randomly assigned to treatment groups.

#### Treatment tolerability

Animals were inspected for changes in general appearance that might occur following a single-dose tolerability assessment prior to the onset of chronic dosing. We monitored coat appearance, piloerection, eye conditions (runny eyes or porphyria, ptosis), gait, tremor, tail tone, posture and reactivity to handling.

Protocols were reviewed and approved by the Institute of Ecology and Biodiversity Institute ethical committee review board, Faculty of Science, University of Chile.

FRAXA-DVI staff blinded to genotypes and drug treatments conducted all experiments. Separate investigators prepared and coded the dosing solutions, allocated the mice to the study treatment groups, dosed the animals and collected the behavioral data.

#### Treatment groups

Two sets of experiments were performed, one with a shorter duration of treatment (Study 1: 5 weeks), and the other with a longer duration (Study 2: 13 weeks) of treatment. All the mice were randomly divided into experimental groups, with 10 mice per group (all at P60). Each set had the following groups:

- Study 1: 5 week treatment with Bryostatin 1.
  - Group 1: the *Fmr1* KO2 mice treated with vehicle,
  - Group 2: the wild-type mice treated with vehicle,
  - Group 3: the *Fmr1* KO2 mice treated with bryostatin-1 for 5 weeks,
  - Group 4: the wild-type mice treated with bryostatin-1 for 5 weeks.
- Study 2: 13 week treatment with Bryostatin 1.
  - Group 1: the *Fmr1* KO2 mice treated with vehicle,
  - Group 2: the wild-type mice treated with vehicle,
  - Group 3: the *Fmr1* KO2 mice treated with bryostatin-1 for 13 weeks,
  - Group 4: the wild-type mice treated with bryostatin-1 for 13 weeks.

Mice were treated with either vehicle or bryostatin-1 (20 µg/m^2^, tail vein i.v., 2 doses/week for either 5 or 13 weeks).

#### Drugs

Bryostatin-1 was dissolved in DMSO (0.1 mg/ml) and kept frozen at – 20 °C. The stock solution was thawed on each day of administration and diluted with DMSO and saline to the used concentration. The same procedure was performed to prepare vehicle control (without bryostatin-1).

### Behavioral and memory tasks

#### Open-field

The apparatus was a gray PVC-enclosed arena 50 × 9 × 30 cm, divided into a 10 cm suares. Mice were moved into the experimental room 5–20 min before testing. A mouse was placed into a corner square facing the corner and observed for 3 min. The movement of the mouse around the field was recorded with a video tracking device for the entire testing period (version NT4.0, Viewpoint). The number of squares entered with the whole body (locomotor activity) were counted and scored as T1 (Time 1). Activity in the open field was measured a second time T2 (10 min after the first testing, Time 2) and a third time T3 (24 h after the first testing, Time 3) to determine a short-term and long-term habituation of the animals to the open field environment.

#### Hippocampal dependent tests of daily living (ADL)

Activities of daily living (ADL) are defined as the things we normally do such as feeding ourselves, bathing and dressing. Deterioration in the ability to perform ADL is an early sign of cognitive decline. Moreover, human episodic memory is different to rodent memory, which seems to be largely non-episodic. Therefore, an approach to preclinical screening would be to characterise the ADL of mice in conjunction with other cognitive tests. In 2005 it was proposed that the hippocampus is vital for the performance of ADL of mice^23^. Rodents with lesions of the hippocampus typically perform very poorly on ADL. Several tests of ADL in the mouse have been developed; nesting, marble burying, hoarding and burrowing^23,24^.

#### Nesting

For small rodents, nests are important in heat conservation as well as reproduction and shelter. Nesting was measured in the home cages of mice. The mice first shred the tightly packed material, then arranged it into a nest. Assigned scores of the quality of the resulting nest: 0, no nest; 1, flat nest; 2, nest covering the mouse. The protocol used pressed cotton squares and a definitive 5-point nest-rating scale. This is a simple, cheap and easily done test that, along with other tests of species-typical behavior, is a sensitive assay for identifying previously unknown behavioral phenotypes^25^. The test was done overnight.

*Nesting scoring* The nests are assessed on a 5-point scale (see Fig. Score: 1–5).

1. The Nestlet is largely untouched (> 90% intact).
2. The Nestlet is partially torn up (50–90% remaining intact).
3. The Nestlet is mostly shredded but often there is no identifiable nest site: < 50% of the Nestlet remains intact but < 90% is within a quarter of the cage floor area, i.e. the cotton is not gathered into a nest but spread around the cage.
4. An identifiable, but flat nest: > 90% of the Nestlet is torn up, the material is gathered into a nest within a quarter of the cage floor area, but the nest is flat, with walls higher than mouse body height (curled up on its side) on less than 50% of its circumference.
5. A (near) perfect nest: > 90% of the Nestlet is torn up, the nest is a crater, with walls higher than mouse body height on more than 50% of its circumference.
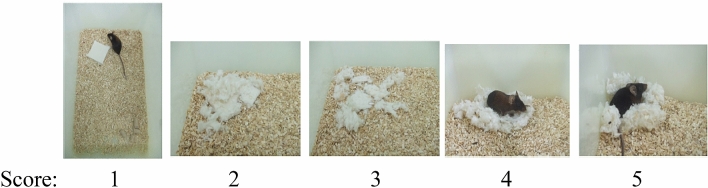

#### Marble burying

Mice exhibit various species-typical behaviors such as digging and burrowing. They dig in the ground to find food, to hoard food, to create a refuge from predators or cold and to make a safe nursery area for the young. In the laboratory, mice dig vigorously in deep bedding such as wood chips. This behavior is sensitive to strain differences and drugs. For example, the effects of anxiolytics and 5-HT-active compounds, including those used clinically for obsessive–compulsive disorder (OCD), can be detected^24–26^. Marble burying is hippocampal dependent. Therefore, one interpretation of marble burying/digging is that it will be affected by any agent affecting hippocampal function, including benzodiazepines and 5-HT active compounds^26^.

Transparent plastic cages are filled with a 10-cm deep layer of sawdust, on top of which 10 glass marbles are placed in two rows. Each animal is left undisturbed in such a cage for 30 min. The number of marbles that are buried to at least two-thirds of their depth is counted^26^.

#### Contextual fear conditioning

Contextual fear conditioning is a cognitive tests and the most basic of the conditioning procedures. It involves taking an animal and placing it in a novel environment, providing an aversive stimulus, and then removing it. Fear conditioning has an emotional component that is generally absent in spontaneous recognition memory tasks. Contextual fear conditioning requires a functional hippocampus and the amygdala circuit. When the animal is returned to the same environment, it generally will demonstrate a freezing response if it remembers and associates that environment with the aversive stimulus. Freezing is a species-specific response to fear, which has been defined as “absence of movement except for respiration.” This may last for seconds to minutes depending on the strength of the aversive stimulus, the number of presentations, and the degree of learning achieved by the subject.

#### Day 1

Program a 120-s habituation period before the first of two identical trials begins. This allows the animal to explore briefly and to take in the aspects of the chamber. A tone (auditory) cue is then presented, generally at a level of 70–80 dB (we use 80 dB) for 15–30 s. A mild foot shock is administered during the last 2 s of the tone presentation and co-terminates with the tone. The foot shock is generally 0.6 mA, (0.17–0.8 mA) for 1–2 s. After the shock presentation, an inter-trial interval (60–210 s) precedes a second identical trial. Following the final shock presentation, the house light should remain on for an additional 60 s, to enable removing the mouse in a 30–60 s time period after the last trial.

#### Day 2

1. Approximately 30 min were allowed before transferring the mouse to a new location for cue testing. 2. The mouse was placed in the chamber and allowed to habituate for 3 min. The same intensity tone cue used in the conditioning session was then activated for the next 3 min. One additional minute of recording without the cue was taken before the animal was removed. Again, the mouse freezing behavior was captured live. Using a Kinder Scientific Motor Monitor, activity beam breaks were recorded and measures of freezing were derived from a computer analysis.

### Statistical analysis

An analysis of variance and normal distribution of the data revealed a not normally distribution (see supplementary material 1). For statistical analysis, the post hoc Mann–Whitney U test was used to evaluate pairwise differences and followed by Kruskal–Wallis test to make groups comparisons (see supplementary material 2).

Data was visualized as boxplots with interquartile ranges, presenting all the data points obtained from the behavioral testing.

## Results

The aim of the study was to evaluate if longer-term treatment with Bryostatin 1 would result in further improvement in FXS phenotypes including repetitive behaviour, hyperactivity and open field habituation, hippocampal dependent ADL and memory in *Fmr1* KO2 mice. Drugs tested in FXS like mGluR5 antagonists, GABA-A and -B agonists show a strong acute effect, but tolerance and little long-term effect occur following chronic treatment. We selected 5 weeks of treatment in a mouse as comparable to many months of human treatment, and 13 weeks of treatment in a mouse as comparable to years of treatment in humans, a consideration in planning possible clinical trials.

### Chronic bryostatin 1 (13 weeks) reduces hyperactivity and rescues habituation to a novel environment in the *Fmr1* KO2 mice

The open field has been widely used for hyperactivity assessment. Habituation to the open field has, however, been used as a means of assessing a simple form of memory (Bolivar, 2009). The open field activity 10 min (second time tested in the open field or T2) and 24 h third time tested in the open field or T3) after the first open field testing (first time tested in the open field or T1), is a simple form of learning (habituation).

**Figure 1:**
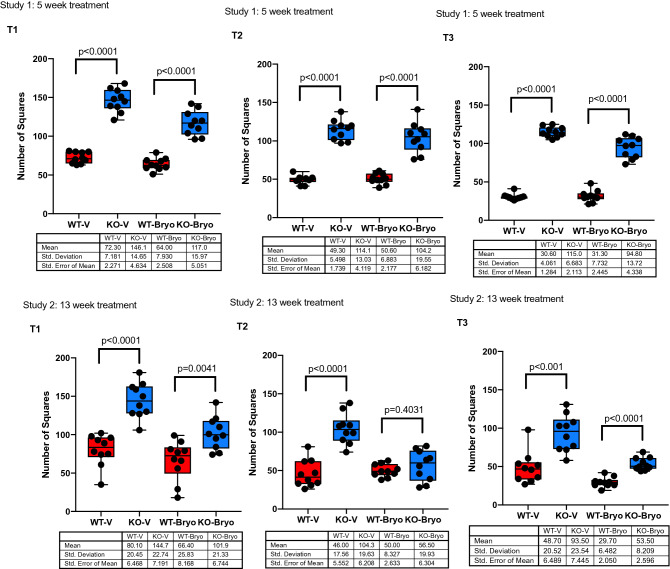
Chronic bryostatin-1 improves the behavioral phenotypes in hyperactivity of the *Fmr1* KO2 mice in an open field test. Wild-type (WT) or *Fmr1* KO2 (KO) adult mice were treated with vehicle (V) or bryostatin 1 (Bryo) for 5 weeks (Study 1) or 13 weeks (Study 2) and then tested in open field. From T1 to T3, data are shown for hyperarousal (Number of Squares crossed in the open field test) (first time tested in the open field or T1), 10 min after the first open field test (second time exposed to the open field or T2), and 24 h after the first open field test (third time exposed to the open field or T3). We identified a significant improvement in Study 2, T 2 in the open field when compared to WT littermates (*P* = 0.4031). Data is visualized as boxplots with interquartile ranges, presenting all the data points. The table indicates the mean standard deviation and the standard error of the mean (N = 10 mice per group).

The *Fmr1* KO2 mice showed approximately twice the level of activity than wild-type (WT) littermates in all open field experiments showing a significant difference among the groups treated with vehicle, for study 1 (5 week treatment with Bryostatin 1) and study 2 (13 week treatment with bryostatin 1) (*P* < 0.0001, Fig. 1). A 5-week treatment with bryostatin 1 (study 1) did not have a positive effect on the activity in the wild-type and *Fmr1* KO2 mice. However, there was a significant amelioration of the hyperactivity phenotype and short term memory improvement in *Fmr1* KO2 mice after 13-week treatment when compared to wild type littermate mice (*P* < 0.4031; Fig. 1 Study 2, T2). Twenty-four hours after the first open field (T3), *Fmr1* KO2 activity in the open field was evaluated as an assessment of habituation and long-term memory (Fig. 1 Study 1 T3 and Fig. 1 Study 2 T3). There was a significant difference among the WT-V and KO-V groups (*P* < 0.0001), treatment with a 5-week bryostatin-1 showed no effect in WT and *Fmr1* KO2 mice showing a significant difference when compared with WT mice (*P* < 0.0001). The open field activity, evaluated 24 h after the first testing, also differed significantly among the groups after a 13-week treatment (*P* < 0.0001; Fig. 1 Study 2 T3).

### Chronic bryostatin-1 (13 weeks) normalizes the nesting behavior in the *Fmr1* KO2 mice

**Figure 2:**
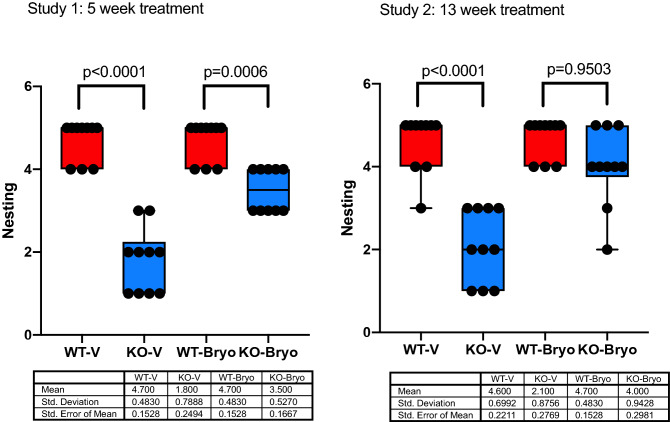
Chronic bryostatin-1 rescues impaired nesting activity of the *Fmr1* KO2 mice. Wild-type (WT) or *Fmr1* KO2 (KO) adult mice were treated with vehicle (V) or bryostatin-1 (Bryo) for 5 weeks (Study 1) or 13 weeks (Study 2) and then tested in nesting activity. Chronic treatment with bryostatin 1 for 13 week improves *Fmr1* KO2 nesting behavior to wild-type levels (*P* = 0.9503). Data is visualized as boxplots with interquartile ranges, presenting all the data points. The table indicates the mean standard deviation and the standard error of the mean (N = 10 mice per group).

Deficits in ADL like nesting behaviour have been observed in models of psychiatric disease, and hippocampal lesions suggesting that hippocampal-like processes could be affecting *Fmr1* KO2 mice and could explain the phenotype^25,26,30,31^. This test has been used as an indicator of hippocampal lesion and dysfunction. WT littermate mice score 4–5 on nest construction and *Fmr1* KO2 a median score around 1–2, a highly significant and a robust phenotype. The results showed a significant difference in the nesting activity between WT-V and KO-V (*P* < 0.0001; Fig. 2 Study 1 and Study 2). Treatment with 5-week bryostatin-1 had no effect on correcting nesting activity in the *Fmr1* KO2 mice when compared with WT littermates (*P* = 0.0006; Fig. 2 Study 1). Interestingly, 13-week treatment with bryostatin-1 resulted in complete normalization of the nesting behavior in *Fmr1* KO2 mice to the level of the wild-type littermate (*P* = 0.9503; Fig. 2 Study 2).

### Chronic bryostatin-1 (13 weeks) normalizes marble burying behavior in the *Fmr1* KO2 mice

**Figure 3:**
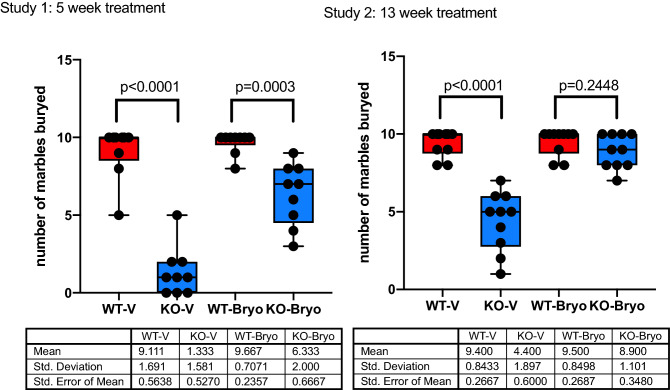
Chronic bryostatin-1 rescues impaired marble burying activity of the *Fmr1* KO2 mice. Wild-type (WT) or *Fmr1* KO2 (KO) adult mice were treated with vehicle (V) or bryostatin-1 (Bryo) for 5 weeks (Study 1) or 13 weeks (Study 2) and then tested in marble burying activity. Thirteen week treatment with Bryostatin 1 significantly improves hippocampal dependent marble burying behavior in *Fmr1* KO2 mice when compared to wild type littermates (*P* = 0.2448). Data is visualized as boxplots with interquartile ranges, presenting all the data points. The table indicates the mean standard deviation and the standard error of the mean (N = 10 mice per group).

Mice spontaneously dig and forage for seeds and food in their natural habitat. Marble burying, an ADL, provides quantitative data on this behavior under controlled laboratory conditions^23,24^. The *Fmr1* KO2 mice in our study buried fewer marbles than their wild-type littermates did. The results showed a significant difference among WT-V and KO-V groups (*P* < 0.0001; Fig. 3 Study 1 and Study 2). A 5-week bryostatin-1 treatment has no therapeutic effect in marble burying activity in the *Fmr1* KO2 mice, keeping significant difference with the WT littermates (*P* = 0.0003). After 13-week treatment with bryostatin-1, marble burying activity in *Fmr1* KO2 mice was rescued to the same level of the wild-type mice (*P* = 0.2448; Fig. 3 Study 2). Importantly, marble burying is not a learned behaviour, but an ADL that does depend on the integrity of the hippocampal circuit.

### Chronic bryostatin-1 (13 weeks) rescues fear learning and memory in the *Fmr1* KO2 mice

**Figure 4:**
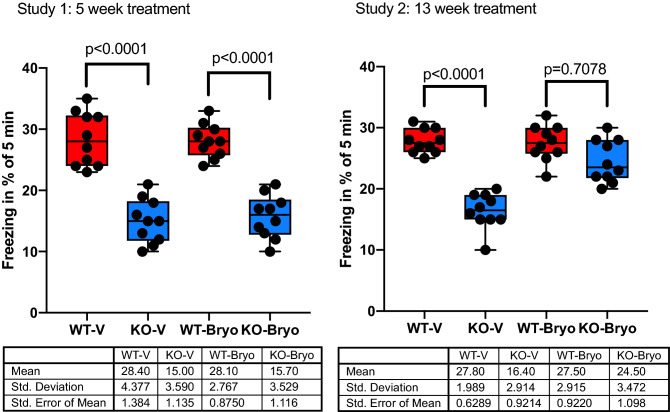
Chronic bryostatin-1 rescues impaired learning and memory of the *Fmr1* KO2 mice in contextual fear conditioning. Wild-type (WT) or *Fmr1* KO2 (KO) adult mice were treated with vehicle (V) or bryostatin-1 (Bryo) for 5 weeks (Study 1) or 13 weeks (Study 2) and then tested in contextual fear conditioning. Significant improvement in cognitive behavior is observe in *Fmr1* KO2 mice following chronic treatment with Bryostatin 1 for 13 week when compared to wild type littermates (*P* = 0.7078). Data is visualized as boxplots with interquartile ranges, presenting all the data points. The table indicates the mean standard deviation and the standard error of the mean (N = 10 mice per group).

Contextual fear conditioning assesses the animal’s ability to make an association between an environmental cue (light, sound) and a paired foot shock, which results in freezing. *Fmr1* KO2 mice showed a significant deficit in contextual fear conditioning memory to an aversive stimulus compared with WT mice. The results showed a significant difference among WT-V and KO-V groups (*P* < 0.0001; Fig. 4 Study 1 and Study 2) in freezing behavior. A 5-week bryostatin-1 treatment has no therapeutic effect in fear learning and memory in the *Fmr1* KO2 mice when compared with WT littermates (*P* < 0.0001) (Fig. 4 Study 1). However, after a 13-week treatment therapeutic impact of bryostatin-1 treatment improved memory retention by enhancing memory acquisition and memory consolidation in *Fmr1* KO2 mice. No significant difference is observed between *Fmr1* KO2 mice and Wild type littermates (*P* = 0.7078) (Fig. 4 Study 2).

## Discussion

The most significant findings of our study are not only that bryostatin-1 can rescue both the phenotypes of autistic and cognitive abnormalities in the *Fmr1* KO2 mice, but also that such a rescue can be achieved only through an appropriate (i.e. chronic) dosing and therapeutic period. A shorter duration of treatment (5-weeks) with the same dose of bryostatin-1 evoked only minimal, partial, and inconsistent effects in the *Fmr1* KO2 mice, while a 13-week treatment with bryostatin-1 rescued all the impaired behaviors evaluated in the *Fmr1* KO2 mice. These include hyperactivity, ADL nesting and marble burying, fear learning and memory, and habituation to a novel environment. It should also be mentioned here that, at this effective dose, chronic bryostatin-1 has been found to produce an anti-depressive effect in rodents^27^, a behavioral abnormality highly associated with autism^28^, though not directly tested in this study. It is possible the mechanism of the delayed therapeutic effect could be similar to anti-depressants that act pharmacologically in days but only therapeutically after 4–6 weeks.

It has been well established that episodic memory is significantly impaired in autism^29^. The *Fmr1* KO2 mice display a spectrum of cognitive and autistic phenotypes and other common natural rodent behaviors, including cognitive impairment, hyperarousal in the open field test, impaired social interaction and depression, reduced activities in building nests and burying marbles^30^. In contrast to other reports^31^ that the *Fmr1* KO2 mice buried more marbles than the WT, however, we consistently observed (in this study and also in another)^30^ that the *Fmr1* KO2 mice exhibited an impaired natural behavior and buried fewer number of the marbles than their age-matched wild-type controls.

The dual effect of bryostatin-1 on phenotypes related to cognitive and autistic abnormalities in the *Fmr1* KO2 mice is rather interesting. A common pathophysiological mechanism may be at the play here, i.e., a failed synaptic response to register experience-mediated cognitive demands or sensory inputs, which is targeted simultaneously by bryostatin-1. Disruptions in experience-dependent information registration (maturation of circuits and synapses) are most likely responsible for tactile defensiveness, a behavioral pattern identified in children with autistic spectrum disorder^20,21^. A delay in the maturation and synaptic connectivity of GABAergic interneurons has also been revealed in the FXS mice, a delay that can be rectified by administration of a TrkB receptor agonist^31,32^. Failure in registering experience-dependent specific spatial representation in the brain and stably maintain it also underlies the spatial learning and memory deficits in the *Fmr1* KO2 mice^33^, resulting from impaired synaptic maturation and communication in the network involved in processing spatial information. The deficits in spatial learning and memory in the *Fmr1* KO2 mice have been shown to be due to neither an impaired exploratory behavior^33^ nor deficits in motivation for an escape^17,18^. An additional behavioral assay, contextual fear conditioning, was also performed in our study to further delineate possible treatment effects. Since this assay targets behaviors regulated by different brain regions from previous studies, the results address the spectrum of activity of bryostatin-1 in rescuing a broad range of phenotypes.

Necessity of developing cognitive and behavioral therapies for ASD and FXS has been repetitively emphasized^34^. Effective therapeutic drugs are needed to improve the life quality of the affected patients, either through activation of the *FMR1* gene^35,36^ or the treatment of the symptoms associated with the disorders. However, agents that increase the FMRP levels or the expression of the *FMR1* gene have not been used in vivo due to safety concerns, such as inducing cellular apoptosis in vivo^36^. Although the CRISPR technique may open new potentials^37^, it also raises many ethical concerns, including often undefined off-target effects. Interestingly, a combination of two chemical modulators of gene activity was reported to be able to stably reactivate the *Fmr1* gene in neurons^38^. The attention deficit hyperactivity disorder (ADHD) symptoms in FXS are currently treated with stimulants, such as preparations of methylphenidates or mixed amphetamine salts, with problems of irritability in young children (5 years or younger)^38,39^. In view of the current lack of specific and effective therapeutics for ASD and FXS, bryostatin-1, with its therapeutic effects on cognitive impairment, hyperactivity, and depression, might have unique therapeutic values for ASD and FXS at its effective dose.

In summary, 13 week treatment with bryostatin 1 demonstrated significant therapeutic effects compared to a 5 week treatment regime. This effect was comparable to other treatment strategies being investigated for FXS. It is possible that longer-term treatment would result in further improvement in fragile X phenotypes. This effect is clearly different from other treatment strategies tested to date, in that the drug shows little acute effect, but strong long-term effects. It also shows no evidence of tolerance, which has been a problem with other drug classes (mGluR5 antagonists, GABA-A and -B agonists). Five weeks of treatment in a mouse is comparable to many months of human treatment, and 13 weeks of treatment in a mouse is comparable to years of treatment in humans and when planning future clinical trials this factor should be considered.

## Supplementary information

Supplementary Information.

## Data Availability

All data generated or analysed during this study are included in this published article (and its Supplementary Information files).

## References

[1] Belmonte MK, Bourgeron T (2006). Fragile X syndrome and autism at the intersection of genetic and neural networks. Nat. Neurosci..

[2] Budimirovic DB, Kaufmann WE (2011). What can we learn about autism from studying fragile X syndrome?. Dev. Neurosci..

[3] Tran SS (2019). Widespread RNA editing dysregulation in brains from autistic individuals. Nat. Neurosci..

[4] Banerjee A (2018). Aberrant RNA translation in fragile X syndrome: From FMRP mechanisms to emerging therapeutic strategies. Brain Res..

[5] Bagni C, Zukin RS (2019). A synaptic perspective of fragile X syndrome and autism spectrum disorders. Neuron.

[6] Jacquemont S, Hagerman RJ, Hagerman PJ, Leehey MA (2007). Fragile-X syndrome and fragile X-associated tremor/ataxia syndrome: two faces of FMR1. Lancet Neurol..

[7] Hagerman RJ (2018). Fragile X-associated neuropsychiatric disorders (FXAND). Front. Psychiatry.

[8] Mullard A (2015). Fragile X disappointments upset autism ambitions. Nat. Rev. Drug Discov..

[9] Berry-Kravis E (2016). Mavoglurant in fragile X syndrome: results of two randomized, double-blind, placebo-controlled trials. Sci. Transl. Med..

[10] Dahlhaus R (2018). Of men and mice: modeling the fragile X syndrome. Front. Mol. Neurosci..

[11] Saré RM (2018). Negative effects of chronic rapamycin treatment on behavior in a mouse model of fragile X syndrome. Front. Mol. Neurosci..

[12] Zhu PJ, Chen CJ, Mays J, Stoica L, Costa-Mattioli M (2018). mTORC2, but not mTORC1, is required for hippocampal mGluR-LTD and associated behaviors. Nat. Neurosci..

[13] Alkon DL, Sun M-K, Nelson TJ (2007). PKC signaling deficits: a mechanistic hypothesis for the origins of Alzheimer’s disease. Trends Pharmacol. Sci..

[14] Nelson TJ (2017). Bryostatin effects on cognitive function and PKCε in Alzheimer’s phase IIa and expanded access trials. J. Alzheimer’s Dis..

[15] Farlow MR (2019). A randomized, double-blind, placebo-controlled, phase II study assessing safety, tolerability, and efficacy of bryostatin in the treatment of moderately seere to severe Alzheimer's disease. J. Alzheimer’s Dis..

[16] Hongpaisan J, Sun M-K, Alkon DL (2011). PKCε activation prevents synaptic loss, Aβ elevation, and cognitive deficits in Alzheimer’s disease transgenic mice. J. Neurosci..

[17] Sun M-K, Hongpaisan J, Lim CS, Alkon DL (2014). Bryostatin-1 restores hippocampal synapses and spatial learning and memory in adult fragile X mice. J. Pharmacol. Exp. Ther..

[18] Sun MK, Hongpaisan J, Alkon DL (2016). Rescue of synaptic phenotypes and spatial memory in young fragile X mice. J. Pharmacol. Exp. Ther..

[19] He CX, Portera-Cailliau C (2013). The trouble with spines in fragile X syndrome: density, maturity and plasticity. Neuroscience.

[20] Padmashri R, Reiner BC, Suresh A, Spartz E, Dunaevsky A (2013). Altered structural and functional synaptic plasticity with motor skill learning in a mouse model of fragile X syndrome. J. Neurosci..

[21] Arroyo ED, Fiole D, Mantri S, Huang CX, Portera-Cailliau C (2019). Dendritic spines in early postnatal Fragile X mice are insensitive to novel sensory experience. J. Neurosci..

[22] Mientjes EJ (2006). The generation of a conditional *Fmr1* knock out mouse model to study Fmrp function in vivo. Neurobiol. Dis..

[23] Deacon RM, Rawlins JN (2005). Hippocampal lesions, species-typical behaviours and anxiety in mice. Behav. Brain Res..

[24] Deacon RM (2006). Housing, husbandry and handling of rodents for behavioral experiments. Nat. Protoc..

[25] Deacon RM (2006). Assessing nest building in mice. Nat. Protoc..

[26] Deacon RM (2006). Digging and marble burying in mice: simple methods for in vivo identification of biological impacts. Nat. Protoc..

[27] Sun M-K, Alkon DL (2013). Cerebral ischemia-induced difference in sensitivity to depression and potential therapeutics in rats. Behav. Pharmacol.

[28] Hudson CC, Hall L, Harkness KL (2019). Prevalence of depressive disorders in individuals with autism spectrum disorder: a meta-analysis. J. Abnorm. Child Psychol..

[29] Wang W (2018). Treating a novel plasticity defect rescues episodic memory in Fragile X model mice. Mol. Psychiatry.

[30] Gurney ME, Cogram P, Deacon RM, Rex C, Tranfaglia M (2017). Multiple behavior phenotypes of the fragile-X syndrome mouse model respond to chronic inhibition of phosphodiesterase-4D (PDE4D). Sci. Rep..

[31] Gholizadeh S, Arsenault J, Xuan IC, Pacey LK, Hampson DR (2014). Reduced phenotypic severity following adeno-associated virus-mediated Fmr1 gene delivery in fragile X mice. Neuropsychopharmacology.

[32] Nomura T (2017). Delayed maturation of fast-spiking interneurons is rectified by activation of the TrkB receptor in the mouse model of fragile x syndrome. J. Neurosci..

[33] Arbab T, Pennartz CMA, Battaglia FP (2018). Impaired hippocampal representation of place in the Fmr1-knockout mouse model of fragile X syndrome. Sci. Rep..

[34] Saldarriaga W (2014). Fragile X syndrome. Colomb Med. (Cali).

[35] Detich N, Bovenzi V, Szyf M (2003). Valproate induces replication-independent active DNA demethylation. J. Biol. Chem..

[36] Bagni C, Tassone F, Neri G, Hagerman R (2012). Fragile X syndrome: causes, diagnosis, mechanisms, and therapeutics. J. Clin. Invest..

[37] Liu XS (2018). Rescue of fragile X syndrome neurons by DNA methylation editing of the FMR1 gene. Cell.

[38] Vershkov D (2019). FMR1 reactivating treatments in fragile X iPSC-derived neural progenitors in vitro and in vivo. Cell Rep..

[39] Hagerman RJ (2009). Advances in the treatment of fragile X syndrome. Pediatrics.

