# Supplementary material for: Chronic bryostatin-1 rescues autistic and cognitive phenotypes in the fragile X mice
Source: Sci Rep. 2020 Oct 22;10:18058. doi: 10.1038/s41598-020-74848-6 (PMC7581799; doi:10.1038/s41598-020-74848-6)
Supplement: Supplementary file 1 — Supplementary Information. [file 41598_2020_74848_MOESM1_ESM.docx]

Chronic bryostatin-1 rescues Autistic and cognitive phenotypes in the fragile X mice

Patricia Cogram^1,2^*, Daniel L, Alkon^3¶^, David Crockford^3¶^, Robert M.J. Deacon^1,2^, Michael J Hurley^4^, Francisco Altimiras^5,6^, Miao-Kun Sun^3^, and Michael Tranfaglia^7^

^1^FRAXA-DVI, FRAXA, Santiago, Chile.

^2^IEB, Faculty of Science, University of Chile, Santiago, Chile.

^3^Neurotrope Bioscience Inc., USA

^4^Neuroimmunology, Biological Sciences, Faculty of Environmental and Life Sciences, University of Southampton, UK.

^5^Faculty of Engineering, Pontificia Universidad Católica de Valparaíso, Valparaíso, Chile.

^6^Faculty of Engineering and Business, Universidad de las Américas, Santiago, Chile.

^7^Michael Tranfaglia, FRAXA Research Foundation, Newburyport, MA, USA

**Supplemantary Information**

| **Groups** | **p-value for Normal distribution (Kolmogorov-Smirnov test)** | | | |
| --- | --- | --- | --- | --- |
|  | WT-V | KO-V | WT-Bryo | KO-Bryo |
| Fear conditioning Study 1 | >0,1000 | >0,1000 | >0,1000 | >0,1000 |
| Fear conditioning Study 2 | >0,1000 | >0,1000 | >0,1000 | >0,1000 |
| Nesting Study 1 | <0,0001 | 0.0913 | <0,0001 | 0.003 |
| Nesting Study 2 | <0,0001 | 0.0819 | <0,0001 | 0.0111 |
| Open field T1 Study 1 | >0,1000 | >0,1000 | >0,1000 | >0,1000 |
| Open field T1 Study 2 | >0,1000 | >0,1000 | >0,1000 | >0,1000 |
| Open field T2 Study 1 | >0,1000 | >0,1000 | >0,1000 | >0,1000 |
| Open field T2 Study 2 | >0,1000 | >0,1000 | >0,1000 | >0,1000 |
| Open field T3 Study 1 | 0.0448 | >0,1000 | >0,1000 | >0,1000 |
| Open field T3 Study 2 | >0,1000 | >0,1000 | >0,1000 | >0,1000 |
| Marble burying Study 1 | 0.0009 | >0,1000 | <0,0001 | >0,1000 |
| Marble burying Study 2 | 0.0006 | >0,1000 | <0,0001 | >0,1000 |

**Table 1.** **Kolmogorov-Smirnov test** was applied for testing if the

behavioural data follows a normal distribution. The observed

distribution is not a normal distribution.

| Groups | **F value for WT vs KO (F-test)** | |
| --- | --- | --- |
|  | WT-V vs KO-V | WT-Bryo vs KO-Bryo |
| Fear conditioning Study 1 | 1.486 | 1.627 |
| Fear conditioning Study 2 | 2.146 | 1.418 |
| Nesting Study 1 | 2.667 | 1.19 |
| Nesting Study 2 | 1.568 | 3.81 |
| Open field T1 Study 1 | 4.165 | 4.057 |
| Open field T1 Study 2 | 1.236 | 1.467 |
| Open field T2 Study 1 | 5.612 | 8.067 |
| Open field T2 Study 2 | 1.25 | 5.732 |
| Open field T3 Study 1 | 2.709 | 3.147 |
| Open field T3 Study 2 | 1.316 | 1.604 |
| Marble burying Study 1 | 1.144 | 8 |
| Marble burying Study 2 | 5.063 | 1.677 |

**Table 2.** **Analysis of variance** (ANOVA) was used to analyze the differences

among the groups

| **Groups** | **p-value for WT vs KO**  **(Mann Whitney test)** | |
| --- | --- | --- |
|  | WT-V vs KO-V | WT-Bryo vs KO-Bryo |
| Fear conditioning Study 1 | <0,0001 | <0,0001 |
| Fear conditioning Study 2 | <0,0001 | 0.0778 |
| Nesting Study 1 | <0,0001 | 0.0006 |
| Nesting Study 2 | <0,0001 | 0.0953 |
| Open field T1 Study 1 | <0,0001 | <0,0001 |
| Open field T1 Study 2 | <0,0001 | 0.0041 |
| Open field T2 Study 1 | <0,0001 | <0,0001 |
| Open field T2 Study 2 | <0,0001 | 0.4031 |
| Open field T3 Study 1 | <0,0001 | <0,0001 |
| Open field T3 Study 2 | 0.0003 | <0,0001 |
| Marble burying Study 1 | <0,0001 | 0.0003 |
| Marble burying Study 2 | <0,0001 | 0.2448 |

**Table 3.** **Mann**–**Whitney U test** was used is used to compare differences between groups with variables not normally distributed.

| Table Analyzed | Fear conditioning Study 1+2 | |  |  |
| --- | --- | --- | --- | --- |
|  |  |  |  |  |
| Kruskal-Wallis test |  |  |  |  |
| P value | <0,0001 |  |  |  |
| Exact or approximate P value? | Approximate |  |  |  |
| P value summary | **** |  |  |  |
|  |  |  |  |  |
| Dunn's multiple comparisons test | Mean rank diff, | Significant? | Summary | P Value |
| WT-V S1 vs. KO-V S1 | 44.95 | Yes | *** | 0.0004 |
| WT-V S1 vs. WT-Bryo S1 | -0.3 | No | ns | >0,9999 |
| WT-V S1 vs. KO-Bryo S1 | 43.25 | Yes | *** | 0.0009 |
| WT-V S1 vs. WT-V S2 | 0.9 | No | ns | >0,9999 |
| WT-V S1 vs. KO-V S2 | 41.3 | Yes | ** | 0.0019 |
| WT-V S1 vs. WT-Bryo S2 | 2.1 | No | ns | >0,9999 |
| WT-V S1 vs. KO-Bryo S2 | 14.2 | No | ns | >0,9999 |
| KO-V S1 vs. WT-Bryo S1 | -45.25 | Yes | *** | 0.0004 |
| KO-V S1 vs. KO-Bryo S1 | -1.7 | No | ns | >0,9999 |
| KO-V S1 vs. WT-V S2 | -44.05 | Yes | *** | 0.0006 |
| KO-V S1 vs. KO-V S2 | -3.65 | No | ns | >0,9999 |
| KO-V S1 vs. WT-Bryo S2 | -42.85 | Yes | ** | 0.001 |
| KO-V S1 vs. KO-Bryo S2 | -30.75 | No | ns | 0.0853 |
| WT-Bryo S1 vs. KO-Bryo S1 | 43.55 | Yes | *** | 0.0008 |
| WT-Bryo S1 vs. WT-V S2 | 1.2 | No | ns | >0,9999 |
| WT-Bryo S1 vs. KO-V S2 | 41.6 | Yes | ** | 0.0017 |
| WT-Bryo S1 vs. WT-Bryo S2 | 2.4 | No | ns | >0,9999 |
| WT-Bryo S1 vs. KO-Bryo S2 | 14.5 | No | ns | >0,9999 |
| KO-Bryo S1 vs. WT-V S2 | -42.35 | Yes | ** | 0.0013 |
| KO-Bryo S1 vs. KO-V S2 | -1.95 | No | ns | >0,9999 |
| KO-Bryo S1 vs. WT-Bryo S2 | -41.15 | Yes | ** | 0.0021 |
| KO-Bryo S1 vs. KO-Bryo S2 | -29.05 | No | ns | 0.1434 |
| WT-V S2 vs. KO-V S2 | 40.4 | Yes | ** | 0.0028 |
| WT-V S2 vs. WT-Bryo S2 | 1.2 | No | ns | >0,9999 |
| WT-V S2 vs. KO-Bryo S2 | 13.3 | No | ns | >0,9999 |
| KO-V S2 vs. WT-Bryo S2 | -39.2 | Yes | ** | 0.0044 |
| KO-V S2 vs. KO-Bryo S2 | -27.1 | No | ns | 0.2525 |
| WT-Bryo S2 vs. KO-Bryo S2 | 12.1 | No | ns | >0,9999 |

**Table 4.** The **Kruskal**-**Wallis test**, nonparametric test, was used to assess for significant differences in the Fear Conditioning test between different treatment groups.

| Table Analyzed | Nesting-Study1+2 |  |  |  |
| --- | --- | --- | --- | --- |
|  |  |  |  |  |
| Kruskal-Wallis test |  |  |  |  |
| P value | <0,0001 |  |  |  |
| Exact or approximate P value? | Approximate |  |  |  |
| P value summary | **** |  |  |  |
|  |  |  |  |  |
| Dunn's multiple comparisons test | Mean rank diff, | Significant? | Summary | P Value |
| WT-V S1 vs. KO-V S1 | 46.6 | Yes | **** | <0,0001 |
| WT-V S1 vs. WT-Bryo S1 | 0 | No | ns | >0,9999 |
| WT-V S1 vs. KO-Bryo S1 | 26.7 | No | ns | 0.2051 |
| WT-V S1 vs. WT-V S2 | 1.7 | No | ns | >0,9999 |
| WT-V S1 vs. KO-V S2 | 43.75 | Yes | *** | 0.0003 |
| WT-V S1 vs. WT-Bryo S2 | 0 | No | ns | >0,9999 |
| WT-V S1 vs. KO-Bryo S2 | 14.85 | No | ns | >0,9999 |
| KO-V S1 vs. WT-Bryo S1 | -46.6 | Yes | **** | <0,0001 |
| KO-V S1 vs. KO-Bryo S1 | -19.9 | No | ns | >0,9999 |
| KO-V S1 vs. WT-V S2 | -44.9 | Yes | *** | 0.0002 |
| KO-V S1 vs. KO-V S2 | -2.85 | No | ns | >0,9999 |
| KO-V S1 vs. WT-Bryo S2 | -46.6 | Yes | **** | <0,0001 |
| KO-V S1 vs. KO-Bryo S2 | -31.75 | Yes | * | 0.04 |
| WT-Bryo S1 vs. KO-Bryo S1 | 26.7 | No | ns | 0.2051 |
| WT-Bryo S1 vs. WT-V S2 | 1.7 | No | ns | >0,9999 |
| WT-Bryo S1 vs. KO-V S2 | 43.75 | Yes | *** | 0.0003 |
| WT-Bryo S1 vs. WT-Bryo S2 | 0 | No | ns | >0,9999 |
| WT-Bryo S1 vs. KO-Bryo S2 | 14.85 | No | ns | >0,9999 |
| KO-Bryo S1 vs. WT-V S2 | -25 | No | ns | 0.3372 |
| KO-Bryo S1 vs. KO-V S2 | 17.05 | No | ns | >0,9999 |
| KO-Bryo S1 vs. WT-Bryo S2 | -26.7 | No | ns | 0.2051 |
| KO-Bryo S1 vs. KO-Bryo S2 | -11.85 | No | ns | >0,9999 |
| WT-V S2 vs. KO-V S2 | 42.05 | Yes | *** | 0.0007 |
| WT-V S2 vs. WT-Bryo S2 | -1.7 | No | ns | >0,9999 |
| WT-V S2 vs. KO-Bryo S2 | 13.15 | No | ns | >0,9999 |
| KO-V S2 vs. WT-Bryo S2 | -43.75 | Yes | *** | 0.0003 |
| KO-V S2 vs. KO-Bryo S2 | -28.9 | No | ns | 0.1036 |
| WT-Bryo S2 vs. KO-Bryo S2 | 14.85 | No | ns | >0,9999 |

**Table 5.** The **Kruskal**-**Wallis test**, nonparametric test, was used to assess for significant differences in the Nesting test between different treatment groups.

| Table Analyzed | Marble burying-Study1+2 | |  |  |
| --- | --- | --- | --- | --- |
|  |  |  |  |  |
| Kruskal-Wallis test |  |  |  |  |
| P value | <0,0001 |  |  |  |
| Exact or approximate P value? | Approximate |  |  |  |
| P value summary | **** |  |  |  |
|  |  |  |  |  |
| Dunn's multiple comparisons test | Mean rank diff, | Significant? | Summary | P Value |
| WT-V S1 vs. KO-V S1 | 44.89 | Yes | *** | 0.0002 |
| WT-V S1 vs. WT-Bryo S1 | -4.889 | No | ns | >0,9999 |
| WT-V S1 vs. KO-Bryo S1 | 26.44 | No | ns | 0.2396 |
| WT-V S1 vs. WT-V S2 | -0.6667 | No | ns | >0,9999 |
| WT-V S1 vs. KO-V S2 | 35.18 | Yes | ** | 0.0093 |
| WT-V S1 vs. WT-Bryo S2 | -2.567 | No | ns | >0,9999 |
| WT-V S1 vs. KO-Bryo S2 | 5.783 | No | ns | >0,9999 |
| KO-V S1 vs. WT-Bryo S1 | -49.78 | Yes | **** | <0,0001 |
| KO-V S1 vs. KO-Bryo S1 | -18.44 | No | ns | >0,9999 |
| KO-V S1 vs. WT-V S2 | -45.56 | Yes | **** | <0,0001 |
| KO-V S1 vs. KO-V S2 | -9.706 | No | ns | >0,9999 |
| KO-V S1 vs. WT-Bryo S2 | -47.46 | Yes | **** | <0,0001 |
| KO-V S1 vs. KO-Bryo S2 | -39.11 | Yes | ** | 0.0019 |
| WT-Bryo S1 vs. KO-Bryo S1 | 31.33 | No | ns | 0.0514 |
| WT-Bryo S1 vs. WT-V S2 | 4.222 | No | ns | >0,9999 |
| WT-Bryo S1 vs. KO-V S2 | 40.07 | Yes | ** | 0.0012 |
| WT-Bryo S1 vs. WT-Bryo S2 | 2.322 | No | ns | >0,9999 |
| WT-Bryo S1 vs. KO-Bryo S2 | 10.67 | No | ns | >0,9999 |
| KO-Bryo S1 vs. WT-V S2 | -27.11 | No | ns | 0.1591 |
| KO-Bryo S1 vs. KO-V S2 | 8.739 | No | ns | >0,9999 |
| KO-Bryo S1 vs. WT-Bryo S2 | -29.01 | No | ns | 0.0863 |
| KO-Bryo S1 vs. KO-Bryo S2 | -20.66 | No | ns | 0.9818 |
| WT-V S2 vs. KO-V S2 | 35.85 | Yes | ** | 0.0048 |
| WT-V S2 vs. WT-Bryo S2 | -1.9 | No | ns | >0,9999 |
| WT-V S2 vs. KO-Bryo S2 | 6.45 | No | ns | >0,9999 |
| KO-V S2 vs. WT-Bryo S2 | -37.75 | Yes | ** | 0.0021 |
| KO-V S2 vs. KO-Bryo S2 | -29.4 | No | ns | 0.0577 |
| WT-Bryo S2 vs. KO-Bryo S2 | 8.35 | No | ns | >0,9999 |

**Table 6.** The **Kruskal**-**Wallis test**, nonparametric test, was used to assess for significant differences in the Marble Burying test between different treatment groups.

| Table Analyzed | Open Field-Study1+2_T1 | |  |  |
| --- | --- | --- | --- | --- |
|  |  |  |  |  |
| Kruskal-Wallis test |  |  |  |  |
| P value | <0,0001 |  |  |  |
| Exact or approximate P value? | Approximate |  |  |  |
| P value summary | **** |  |  |  |
|  |  |  |  |  |
| Dunn's multiple comparisons test | Mean rank diff, | Significant? | Summary | P Value |
| WT-V S1 vs. KO-V S1 | -46.75 | Yes | *** | 0.0002 |
| WT-V S1 vs. WT-Bryo S1 | 9.6 | No | ns | >0,9999 |
| WT-V S1 vs. KO-Bryo S1 | -31.5 | No | ns | 0.0681 |
| WT-V S1 vs. WT-V S2 | -6.95 | No | ns | >0,9999 |
| WT-V S1 vs. KO-V S2 | -44.9 | Yes | *** | 0.0004 |
| WT-V S1 vs. WT-Bryo S2 | 1.4 | No | ns | >0,9999 |
| WT-V S1 vs. KO-Bryo S2 | -22.1 | No | ns | 0.9361 |
| KO-V S1 vs. WT-Bryo S1 | 56.35 | Yes | **** | <0,0001 |
| KO-V S1 vs. KO-Bryo S1 | 15.25 | No | ns | >0,9999 |
| KO-V S1 vs. WT-V S2 | 39.8 | Yes | ** | 0.0036 |
| KO-V S1 vs. KO-V S2 | 1.85 | No | ns | >0,9999 |
| KO-V S1 vs. WT-Bryo S2 | 48.15 | Yes | *** | 0.0001 |
| KO-V S1 vs. KO-Bryo S2 | 24.65 | No | ns | 0.495 |
| WT-Bryo S1 vs. KO-Bryo S1 | -41.1 | Yes | ** | 0.0021 |
| WT-Bryo S1 vs. WT-V S2 | -16.55 | No | ns | >0,9999 |
| WT-Bryo S1 vs. KO-V S2 | -54.5 | Yes | **** | <0,0001 |
| WT-Bryo S1 vs. WT-Bryo S2 | -8.2 | No | ns | >0,9999 |
| WT-Bryo S1 vs. KO-Bryo S2 | -31.7 | No | ns | 0.0639 |
| KO-Bryo S1 vs. WT-V S2 | 24.55 | No | ns | 0.5081 |
| KO-Bryo S1 vs. KO-V S2 | -13.4 | No | ns | >0,9999 |
| KO-Bryo S1 vs. WT-Bryo S2 | 32.9 | Yes | * | 0.0432 |
| KO-Bryo S1 vs. KO-Bryo S2 | 9.4 | No | ns | >0,9999 |
| WT-V S2 vs. KO-V S2 | -37.95 | Yes | ** | 0.0073 |
| WT-V S2 vs. WT-Bryo S2 | 8.35 | No | ns | >0,9999 |
| WT-V S2 vs. KO-Bryo S2 | -15.15 | No | ns | >0,9999 |
| KO-V S2 vs. WT-Bryo S2 | 46.3 | Yes | *** | 0.0002 |
| KO-V S2 vs. KO-Bryo S2 | 22.8 | No | ns | 0.7901 |
| WT-Bryo S2 vs. KO-Bryo S2 | -23.5 | No | ns | 0.6642 |

**Table 7.** The **Kruskal**-**Wallis test**, nonparametric test, was used to assess for significant differences in the Open Field (T1) test between different treatment groups.

| Table Analyzed | Open Field-Study1+2_T2 | |  |  |
| --- | --- | --- | --- | --- |
|  |  |  |  |  |
| Kruskal-Wallis test |  |  |  |  |
| P value | <0,0001 |  |  |  |
| Exact or approximate P value? | Approximate |  |  |  |
| P value summary | **** |  |  |  |
|  |  |  |  |  |
| Dunn's multiple comparisons test | Mean rank diff, | Significant? | Summary | P Value |
| WT-V S1 vs. KO-V S1 | -43.05 | Yes | *** | 0.001 |
| WT-V S1 vs. WT-Bryo S1 | -1.3 | No | ns | >0,9999 |
| WT-V S1 vs. KO-Bryo S1 | -38.05 | Yes | ** | 0.007 |
| WT-V S1 vs. WT-V S2 | 6.1 | No | ns | >0,9999 |
| WT-V S1 vs. KO-V S2 | -37.45 | Yes | ** | 0.0087 |
| WT-V S1 vs. WT-Bryo S2 | 0.3 | No | ns | >0,9999 |
| WT-V S1 vs. KO-Bryo S2 | -5.35 | No | ns | >0,9999 |
| KO-V S1 vs. WT-Bryo S1 | 41.75 | Yes | ** | 0.0016 |
| KO-V S1 vs. KO-Bryo S1 | 5 | No | ns | >0,9999 |
| KO-V S1 vs. WT-V S2 | 49.15 | Yes | **** | <0,0001 |
| KO-V S1 vs. KO-V S2 | 5.6 | No | ns | >0,9999 |
| KO-V S1 vs. WT-Bryo S2 | 43.35 | Yes | *** | 0.0008 |
| KO-V S1 vs. KO-Bryo S2 | 37.7 | Yes | ** | 0.008 |
| WT-Bryo S1 vs. KO-Bryo S1 | -36.75 | Yes | * | 0.0113 |
| WT-Bryo S1 vs. WT-V S2 | 7.4 | No | ns | >0,9999 |
| WT-Bryo S1 vs. KO-V S2 | -36.15 | Yes | * | 0.014 |
| WT-Bryo S1 vs. WT-Bryo S2 | 1.6 | No | ns | >0,9999 |
| WT-Bryo S1 vs. KO-Bryo S2 | -4.05 | No | ns | >0,9999 |
| KO-Bryo S1 vs. WT-V S2 | 44.15 | Yes | *** | 0.0006 |
| KO-Bryo S1 vs. KO-V S2 | 0.6 | No | ns | >0,9999 |
| KO-Bryo S1 vs. WT-Bryo S2 | 38.35 | Yes | ** | 0.0062 |
| KO-Bryo S1 vs. KO-Bryo S2 | 32.7 | Yes | * | 0.0461 |
| WT-V S2 vs. KO-V S2 | -43.55 | Yes | *** | 0.0008 |
| WT-V S2 vs. WT-Bryo S2 | -5.8 | No | ns | >0,9999 |
| WT-V S2 vs. KO-Bryo S2 | -11.45 | No | ns | >0,9999 |
| KO-V S2 vs. WT-Bryo S2 | 37.75 | Yes | ** | 0.0078 |
| KO-V S2 vs. KO-Bryo S2 | 32.1 | No | ns | 0.056 |
| WT-Bryo S2 vs. KO-Bryo S2 | -5.65 | No | ns | >0,9999 |

**Table 8.** The **Kruskal**-**Wallis test**, nonparametric test, was used to assess for significant differences in the Open Field (T2) test between different treatment groups.

| Table Analyzed | Open Field-Study1+2_T3 | |  |  |
| --- | --- | --- | --- | --- |
|  |  |  |  |  |
| Kruskal-Wallis test |  |  |  |  |
| P value | <0,0001 |  |  |  |
| Exact or approximate P value? | Approximate |  |  |  |
| P value summary | **** |  |  |  |
|  |  |  |  |  |
| Dunn's multiple comparisons test | Mean rank diff, | Significant? | Summary | P Value |
| WT-V S1 vs. KO-V S1 | -55.4 | Yes | **** | <0,0001 |
| WT-V S1 vs. WT-Bryo S1 | -1.2 | No | ns | >0,9999 |
| WT-V S1 vs. KO-Bryo S1 | -43.65 | Yes | *** | 0.0007 |
| WT-V S1 vs. WT-V S2 | -18.15 | No | ns | >0,9999 |
| WT-V S1 vs. KO-V S2 | -43.65 | Yes | *** | 0.0007 |
| WT-V S1 vs. WT-Bryo S2 | 2.35 | No | ns | >0,9999 |
| WT-V S1 vs. KO-Bryo S2 | -24.7 | No | ns | 0.4876 |
| KO-V S1 vs. WT-Bryo S1 | 54.2 | Yes | **** | <0,0001 |
| KO-V S1 vs. KO-Bryo S1 | 11.75 | No | ns | >0,9999 |
| KO-V S1 vs. WT-V S2 | 37.25 | Yes | ** | 0.0094 |
| KO-V S1 vs. KO-V S2 | 11.75 | No | ns | >0,9999 |
| KO-V S1 vs. WT-Bryo S2 | 57.75 | Yes | **** | <0,0001 |
| KO-V S1 vs. KO-Bryo S2 | 30.7 | No | ns | 0.0874 |
| WT-Bryo S1 vs. KO-Bryo S1 | -42.45 | Yes | ** | 0.0012 |
| WT-Bryo S1 vs. WT-V S2 | -16.95 | No | ns | >0,9999 |
| WT-Bryo S1 vs. KO-V S2 | -42.45 | Yes | ** | 0.0012 |
| WT-Bryo S1 vs. WT-Bryo S2 | 3.55 | No | ns | >0,9999 |
| WT-Bryo S1 vs. KO-Bryo S2 | -23.5 | No | ns | 0.6629 |
| KO-Bryo S1 vs. WT-V S2 | 25.5 | No | ns | 0.3946 |
| KO-Bryo S1 vs. KO-V S2 | 0 | No | ns | >0,9999 |

**Table 9.** The **Kruskal**-**Wallis test**, nonparametric test, was used to assess for significant differences in the Open Field (T3) test between different treatment groups.
